# Penthorum Chinense Pursh Extract Alleviates Aflatoxin B1-Induced Liver Injury and Oxidative Stress Through Mitochondrial Pathways in Broilers

**DOI:** 10.3389/fvets.2022.822259

**Published:** 2022-02-02

**Authors:** Fazul Nabi, Weilai Tao, Ruiling Ye, Zhenzhen Li, Qin Lu, Yangfei Shang, Yu Hu, Jiali Fang, Zohaib Ahmed Bhutto, Juan Liu

**Affiliations:** ^1^Department of Traditional Chinese Veterinary Medicine, College of Veterinary Medicine, Southwest University, Chongqing, China; ^2^Department of Poultry Science, Faculty of Veterinary and Animal Science, Lasbela University of Agriculture, Water and Marine Sciences, Uthal, Pakistan; ^3^Chinese Veterinary Herbal Drugs Innovation Research Laboratory, University Veterinary Science Engineering Research Center in Chongqing, Chongqing, China; ^4^Immunology Research Center of Medical Research Institute, Southwest University, Chongqing, China

**Keywords:** penthorum chinense pursh extract, liver, broilers, aflatoxin B1, apoptosis

## Abstract

Aflatoxin is an important toxicant of the fungal origin and poses a threat to the poultry industry. This study was designed to reveal the underlying mechanism and protective methods against aflatoxin B1 (AFB1)-induced liver injury, oxidative stress, and apoptosis using a Traditional Chinese medicine, Penthorum chinense Pursh extract (PCPE), in broilers. A total of 164 (day-old) broilers were equally allocated to the control, AFB1 (3 mg/kg feed), positive drug (Yin-Chen-Hao Tang extract, 10 ml/kg feed), PCPE (2 g PCPE/kg), and PCPE low, medium, and high dose groups (1 g, 2 g, 3 g PCPE/kg feed, respectively). AFB1 significantly decreased the growth performance and serum immunoglobulin level, altered normal serum biochemical parameters and antioxidant activities, and induced histopathological lesions in the liver as compared to control group. Additionally, AFB1 significantly up-regulated the mRNA expression levels of apoptosis-related genes such as Bax, Bak, caspase-9, caspase-3, and p53, whereas it down-regulated the expression levels of BCL2 in the liver of broilers. The supplementation of different doses of PCPE to AFB1-affected birds significantly eased AFB1 negative effects by improving growth performance, immunoglobulin level, and oxidative capacity, and reversed oxidative stress and pathological lesions in liver. Furthermore, supplementation of PCPE to the AFB1 group reversed apoptosis by significantly down-regulating the mRNA expression levels of Bax, Bak, caspase-9, caspase-3, and p53 and up-regulating the expression levels of BCL2 in the liver of broilers. Based on these results, we conclude that supplementation of PCPE is protective and safe against oxidative stress, is anti-apoptotic, and reverses the liver damage caused by AFB1 in broilers.

## Introduction

Mycotoxins produced by *Aspergillus* fungi are widespread and potent type of toxicants in the poultry industry globally. Aflatoxin B1 (AFB1) is a serious threat to the poultry industry and poses a great risk to public health (1, 2). Natural or artificial AFB1-contaminated feed results in aflatoxicosis in poultry, that can subsequently lower the growth performance and immunity in broilers (3). AFB1 toxicity has been widely studied in humans and animals for their adverse effects, such as hepatotoxic, immunotoxic, carcinogenic, mutagenic, teratogenic, and other adverse health effects on several vital organs (4, 5). AFB1 is the hazardous and commonly occurring mycotoxin in poultry which negatively impact on productivity and high susceptibility to pathogenicity in poultry (6). Aflatoxin affect on several vital organs including spleen, kidney, thymus, bursa of Fabricius among them liver is mostly affected that causes macroscopic and microscopic liver changes (3).

The liver is a vital organ with numerous functions in broilers; however, it is also the main target organ of AFB1 where aflatoxins are metabolized and converted into extremely toxic forms, thereby invading the liver, and resulting in severe hepatotoxicity. AFB1 destroys the normal structure of hepatocytes and mitochondria, subsequently altering the antioxidant system. Furthermore, autophagy eliminates impaired cellular structures and apoptosis is initiated by liver hepatocytes to maintain liver function; however, it also causes hepatotoxicity (7–9). The metabolic and toxic effects of AFB1 are principally observed in liver tissues, and previous studies have suggested that hepatic cell apoptosis leads to liver damage in poultry.

Apoptosis is the programmed cell death phenomenon which is essential for normal tissue homeostasis, and it is also associated with the development of several pathogenic diseases in animals (10, 11). In experimental models, apoptosis is performed for the validation of interventions in animals; aflatoxins induce apoptosis *via* cellular toxicity, and inhibition of carbohydrate and lipid metabolism and protein synthesis (12). In poultry, AFB1 can severely alter immunity, cause oxidative damage, and induce apoptosis and development of histopathological lesions in lymphoid tissues. Additionally, AFB1 exposure may alter the size of immune organs, thereby acclimatizing it to stress and severely altering the immune functions in broilers (13–15). Studies have reported that AFB1 can lead to renal injury, respiratory diseases, neuropathy, and liver damages through the induction of oxidative stress and apoptosis.

In the recent years, traditional Chinese medicine (TCM) or ethnomedicine is an emerging discipline. TCM is a systematic methodology for identifying various pathological biomarkers, evaluating the efficiency of herbal medicine, and finding the material basis of herbal formulas. Penthorum chinense Pursh (PCP) is a well-known TCM herbal medicine, and its main extract or ingredient has antioxidant, anti-cancer, and anti-apoptotic activities. Protection against infectious hepatitis and edema, and treatment of various liver diseases are the main functions of PCP (16–19). Our previous study showed that treatment using a PCP compound protected kidney cells from excessive apoptosis by inhibiting the mitochondrial apoptosis pathway activated by AFB1 (19). Therefore, this study was undertaken to further examine the ameliorative effects of PCP extract on oxidative stress and apoptosis through mitochondrial pathways in mycotoxin-mediated toxicity in the liver of broilers.

## Materials and Methods

### Preparation of PCP Extract

The whole PCP grass was provided by Gulin County, Luzhou City, Sichuan Province, China. The botanical origin was identified by Professor Liu Juan, College of Veterinary Medicine, Southwest University, Chongqing, China. The whole grass was cut into small pieces, decocted with 4000 ml ethanol solution (40%), followed by reflux extraction for 120 min. The filtrate thus obtained was decompressed in a rotary vacuum evaporator at 70 °C, and subsequently dried by blowing at 30 °C to obtain a PCPE powder.

### Preparation of Positive Drug Yin-Chen-Hao Tang Extract

YCHT was prepared according to the protocol used in our previous experiment (19). Briefly, Yin chen (54 g) was mixed in 3.6 L deionized water and boiled. The solution was reduced up to 1.8 L, and then samples of gardenia (Gardenia jasminoides) and rhubarb (Rheum officinal baill) (27 and 18 g, respectively) were mixed. Total solution was again boiled for 30 min and was finally filtered. Next, the filtrate was concentrated up to 100 ml by vacuum (equivalent to 1 g herb ml ^1^) and stored at 4°C.

### Bird Grouping and Sample Collection

All experimental procedures agreed with the animal ethics regulations and were accepted by the Institutional Animal Care and Use Committee (IACUC) of Southwest University (IACUC-20201203). Cobb broilers (total 164) 1-day-old) were bought from a commercial hatchery (Daan, Zigong, Sichuan, China). Broilers were housed with standard hygienic and optimal conditions (temperature: 28–30°C, relative humidity (RH): 60–70%). After 7 days of acclimatization period, the broilers were equally divided in the following groups (*n* = 24 per group) with four replicates: control, AFB1, positive drug (YCHT), PCPE (2 g/kg feed), and PCPE high (3 g/kg feed), PCPE medium (2 g/kg feed), and PCPE low-dose (1 g/kg feed) groups. These herbal doses were randomly selected for the experiment. All groups were fed with AFB1 (produced by *Aspergillus flavus* NRRL3357) at 3 mg/kg feed while the positive drug group (YCHT) was fed with 8 ml/kg feed, except the control group till the research trial ended (28 days). All birds were weighed, the weekly average growth parameters, mortality, and morbidity were recorded during the experiment (day 7, 14, 21, 28). Birds were slayed by cervical dislocation; and blood and liver sampling were performed on the 14^th^ and 28^th^ day of the research trial for subsequent analysis.

### Analysis of Antioxidants and Oxidative Biomarkers in the Liver and Serum

Aspartate aminotransferase (AST), alanine transaminase (ALT), alkaline phosphatase (ALP), albumin (ALB), and total bilirubin (TBIL) induced in the serum were quantified using a biochemical analyzer, in accordance with manufacturer's guidelines (Beckman Instruments Inc, USA). Malondialdehyde (MDA), superoxide dismutase (SOD), catalase (CAT), glutathione (GSH), and glutathione peroxidase (GSH-Px) activities in the liver homogenate were measured by enzyme-linked immunosorbent assay (ELISA) following specific kit instructions (Xiamen Jiahui Biotechnology Co. Ltd, Xiamen, China).

### Liver Histology

Hematoxylin and eosin (H and E) staining was performed on the liver tissues. In brief, the tissue samples were fixed in 10% formaldehyde for 24 h and placed in running water overnight. Dehydration was performed using ethanol, clearing using xylene, and embedding using paraffin wax. The sections were incised in 4–5 μm size for the preparation of slides and stained with H and E. Histopathological changes in the liver were examined under an optical microscope (Axio Scope, Germany).

### Reverse Transcription Quantitative Real-Time Polymerase Chain Reaction

The liver tissue samples were collected from each group on day 14 and 28 of the experiment for RT-qPCR analysis. PCR was performed by applying primers for individual concerned genes (Table 1). Total RNA extractions, cDNA synthesis, and RT-qPCR were performed according to the methods used in our previous experiments (19). The collected liver tissue sample (2–3 gm) homogenates were prepared by tissue homogenizer (T10 Basic ULTRA-TURAX ® Germany) in liquid nitrogen along with TRIzol reagent for the extraction of total RNA (1 ml/50 mg liver tissue) (Win Biosciences, Beijing, China). cDNA was synthesized using the Trans Gen cDNA kit (Biotech Co., Ltd., Beijing, China) following the protocol in a 20 μl reaction mixture (oligo (dT)^18^, 2 × TS reaction mix, and 5 μg RNA), and reverse transcription was executed at 42°C for 60 min and 95°C for 3 min. The mRNA expression of genes encoding B-cell lymphoma 2 (*BCL2*), P53 (*P53*), BAX (*BAX)*, BAK (*BAK1*), Caspase-9 (*CASP9*), and Caspase-3 (*CASP3*) was analyzed *via* SYBR Green I real-time fluorescent quantitative PCR system. All reactions were run at least in quadruplicate using the TransStart Green qPCR SuperMix kit (TransGen Biotech, Beijing, China) in a total 20 μl reaction volume containing 2 μl cDNA, 0.5 μl sense and antisense primers, and 10 μl SYBR Green qPCR dye with following parameters: 94°C for 30 s, 40 amplification cycles at 94°C for 5 s, 61°C for 35 s, and 72°C for 30 s. Relative quantification of each gene was performed using the comparative 2^−ΔΔCT^ method.

**Table 1:** Primers for quantitative real-time PCR (19).

**Target gene**	**Primer sequence (5^′^-3^′^)**	**Product length (bp)**
Bcl-2	F: CTGGATCCAGGACAACGGA	19
	R: GATGCAAGCTCCCACCAGAA	20
Caspase-3	F: GAAGATCACAGCAAGCGAAGC	21
	R: CAAGAGGGCCATCTGTACCAT	21
Caspase-9	F: CCGGAGGGATTTATGGAACAG	21
	R: CAGGCCTGGATGAAGAAGAGT	21
P53	F: GTCCCATCCACGGAGGATTAT	21
	R: CCAGGCGGCAATAGACCTTA	20
Bak	F: GGCCATCACGAGAGATCAATG	21
	R: TCCTGTTGGTAGCGGTAGAAG	21
Bax	F: CAGATTGGAGAGGCCCTCTT	20
	R: AATCTGGTCCTGGCTGTTGC	20
GAPDH	F: CAGAACATCATCCCAGCGTC	20
	R: GGCAGGTCAGGTCAACAAC	19

### Inflammatory Mediators and Chicken Immunoglobulins Analysis in Serum

The blood serum content [interleukin (IL)-1, IL-10] was determined on day 14 and 28. Chicken immunoglobulins (IgA, IgM, and IgG) in serum was analyzed by ELISA according to the manufacture's kit instructions (Xiamen Jiahui Biotechnology Co. Ltd., Xiamen, China).

### Statistical Analyses

The data were analyzed using one-tailed analysis of variance using the SPSS software, version 20.0.0 (IBM, Armonk, NY, USA), followed by Duncan's multiple-range test. Data are represented as mean ± standard error (S.E.). Graph Pad Prism (San Diego, CA, USA) was used to generate graphs with error bars.

## Results

### Serum Biochemistry

**Figure 1:**
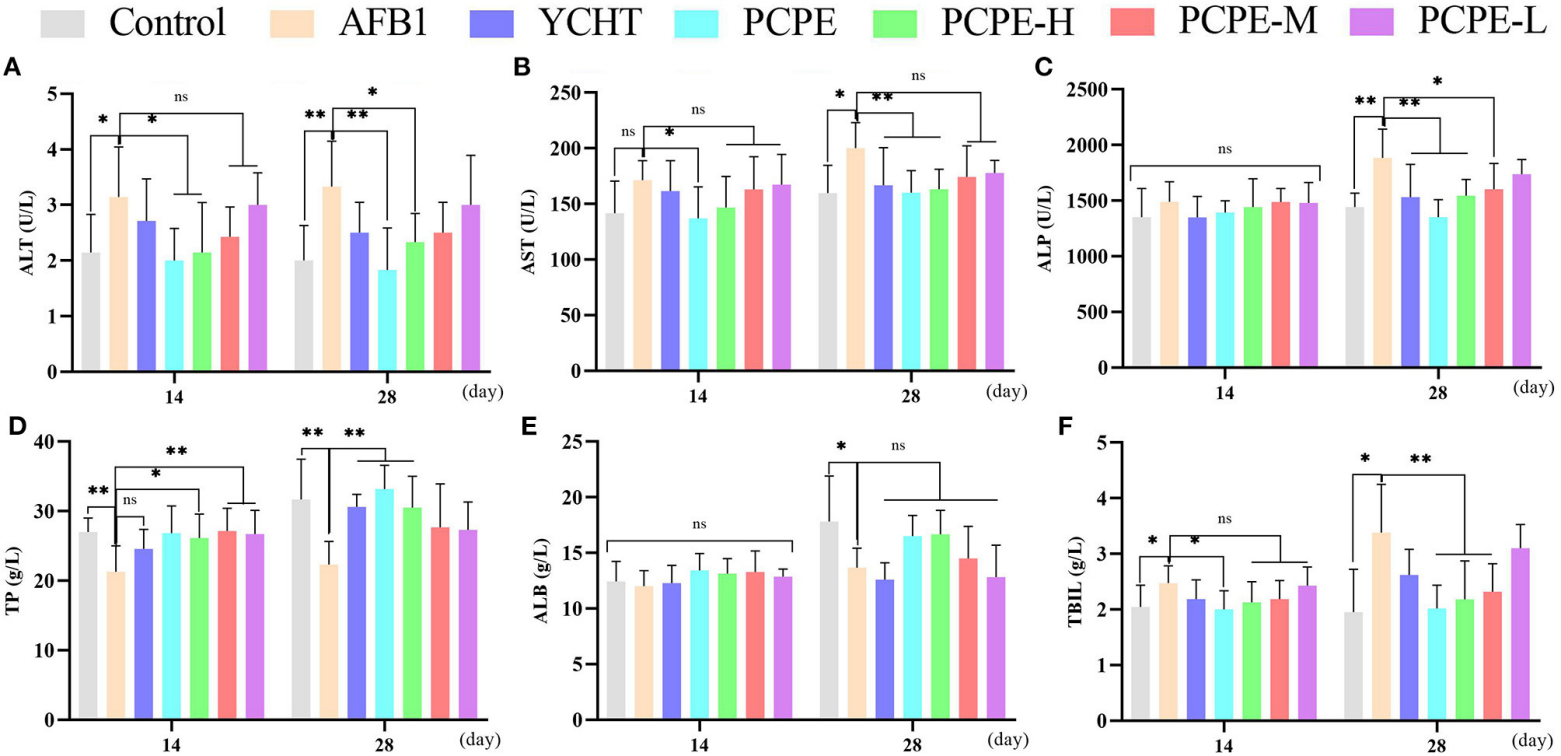
Effect of PCPE on serum biochemistry of broilers feed containing AFB1 and PCPE low, medium, and high dose (1 g, 2 g, 3 g PCPE/kg feed, respectively). Values are represented as the mean ± SD. **(A)** ALT; **(B)** AST; **(C)** ALP; **(D)** TP; **(E)** ALB; **(F)** TBIL. *P < 0.05; **P < 0.01.

The effect of different doses of PCPE and AFB1 lead to biochemical changes in serum revealed that feed contaminated with AFB1 produced an increase in ALT, AST, ALP, and TBIL indices and decreased the total protein (TP) and ALB indices on day 14 and 28 as compared with the control. Compared to the AFB1-affected group with no PCPE administration, AFB1-affected groups administered with different doses of PCPE and positive drug showed restored biochemical changes on day 28 and a significant reversion of these levels. The most ameliorated beneficial effects were found in PCPE alone and high dose groups (3 g/kg feed). However, PCPE produced no impact on biochemical indices on day 14 when compared with the control (Figure 1).

### Histopathology of Liver

**Figure 2:**
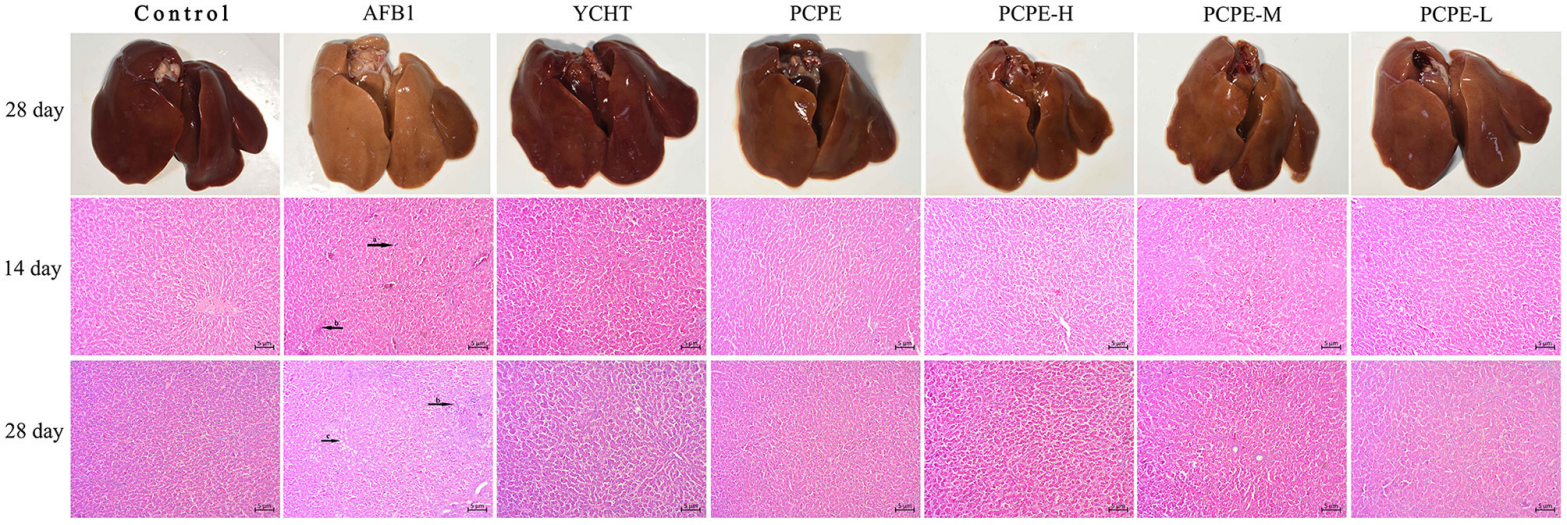
Histopathological analysis of liver from different groups on day 14 and 28 in different groups of the experiment. (a) intrahepatic hemorrhages, (b) inflammatory cells infiltration and fatty degenerations, (c) bile duct hyperplasia.

The effect of PCPE and AFB1 on the liver tissues histopathology was observed in broilers on day 14 and 28 in all groups. When compared with the control group, significant liver damage was observed in the AFB1 group, including intrahepatic hemorrhages, inflammatory cell infiltration and fatty degenerations, and bile duct hyperplasia of broilers. However, the supplementation of PCPE and positive drug (YCHT) to AFB1 diets inhibited the damage to the hepatic parenchyma of liver, especially in alone and high dose PCPE groups (3 g/kg feed) (Figure 2).

### Serum Antioxidant Parameters

**Figure 3:**
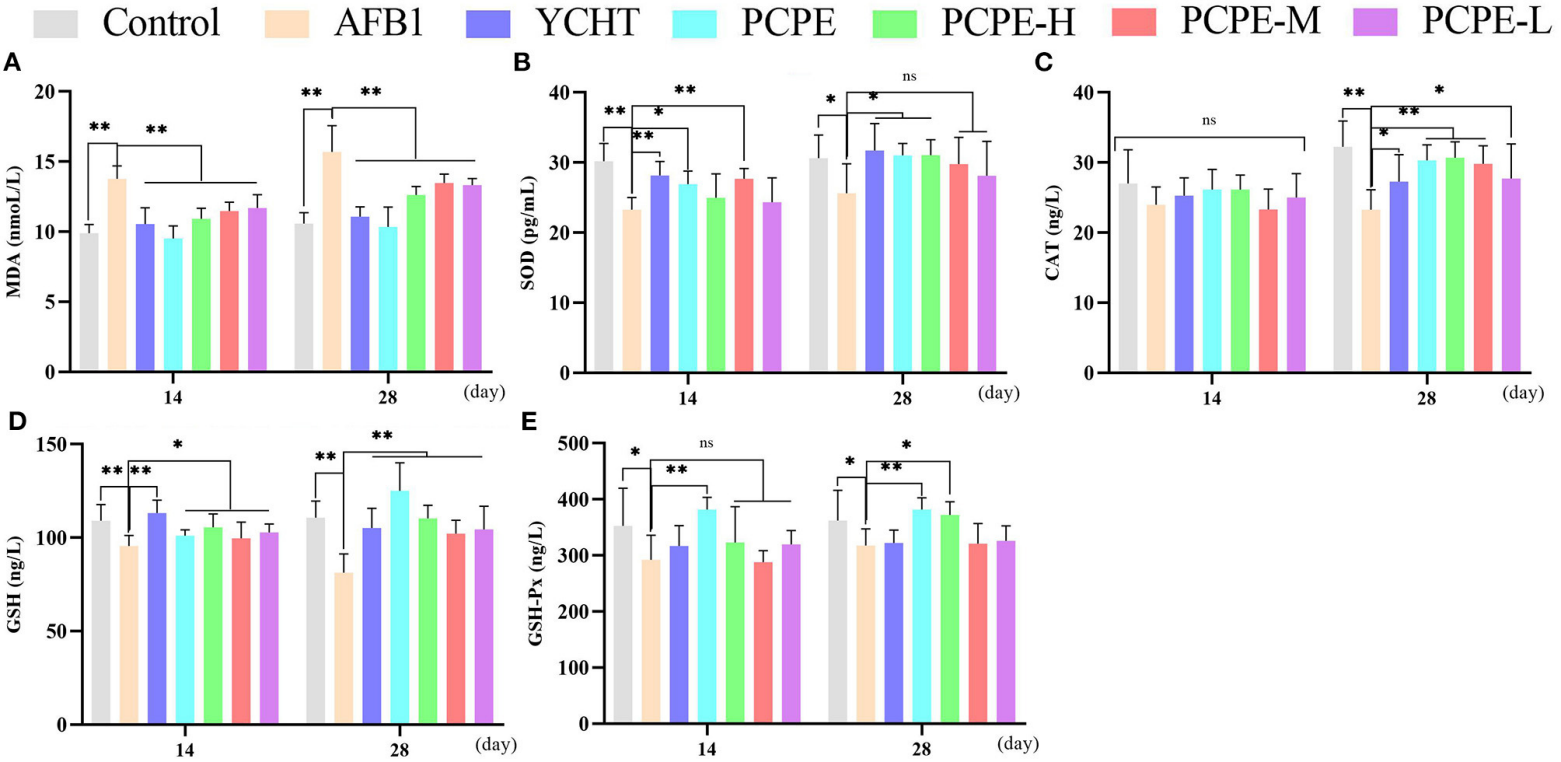
Effect of PCPE on serum antioxidant parameters. Values are represented as mean ± SD. Mean values were significantly different (*P < 0.05; **P < 0.01), ns, non-significant. **(A)** MDA **(B)** SOD **(C)** CAT **(D)** GSH **(E)** GSH-Px.

The effect of different doses of PCPE and AFB1 on serum antioxidant levels on day 14 and 28 revealed that AFB1 increased serum MDA and decreased the activities and concentrations of SOD and CAT and GSH and GSH-Px (*p* < 0.05), respectively, as compared with the control group. However, the addition of different doses of PCPE and positive drug (YCHT) into diets significantly improved the antioxidant activities in serum by decreasing MDA and increasing SOD, CAT, GSH, and GSH-Px. The highly beneficial results were significantly found in PCPE alone and high dose (3 mg/kg feed) groups on 28^th^ day (Figure 3).

### mRNA Expressions of Apoptotic Genes in the Liver

**Figure 4:**
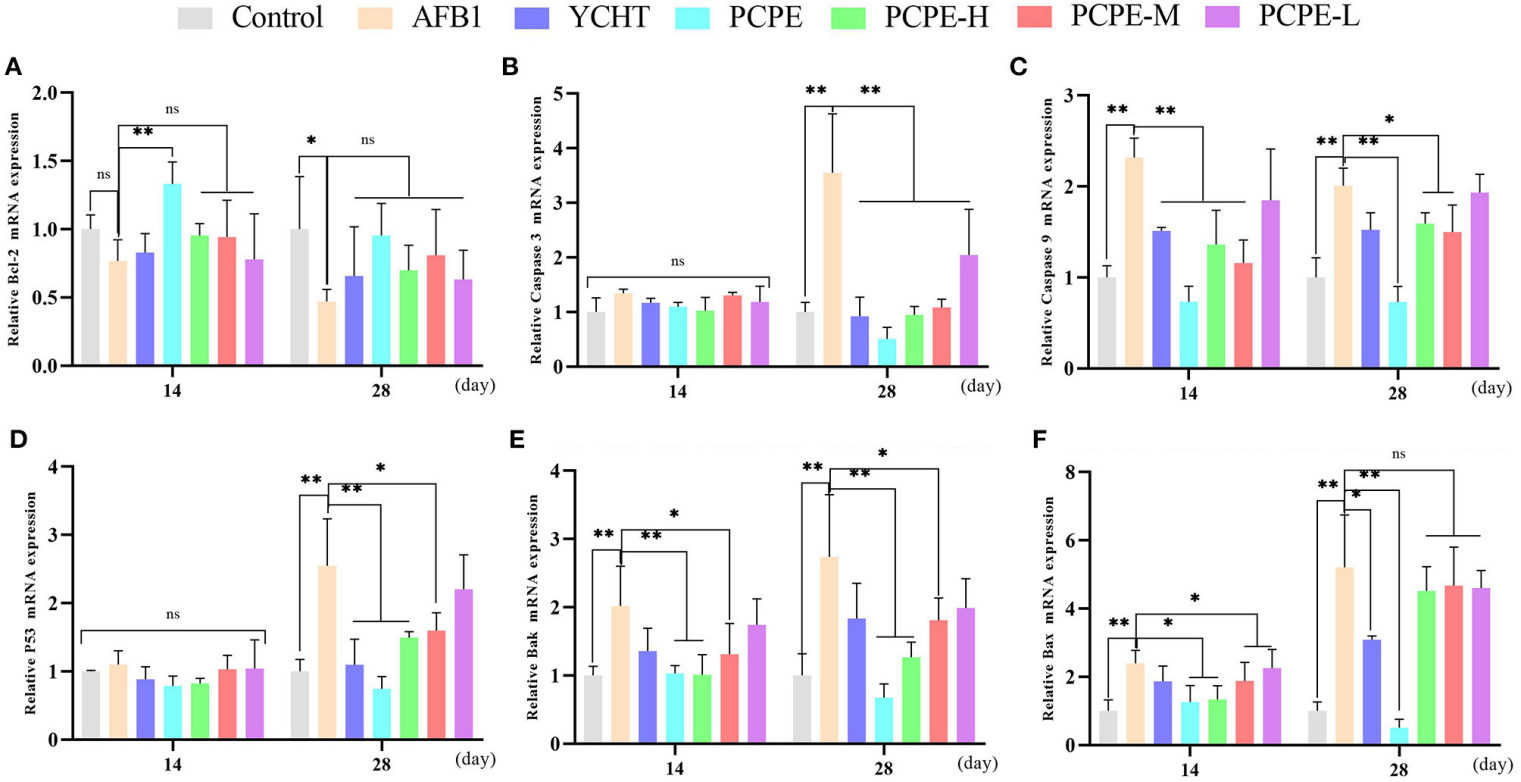
Effect of PCPE on mRNA expressions of apoptosis-genes in liver of Broilers. The mRNA expressions levels of apoptosis-genes were identified by real-time PCR. All data were expressed as mean ± SD. Asterisk means significant difference (*p* < 0.05) compared to the control and AFB1 group. **(A)** Bcl-2; **(B)** Caspase-3; **(C)** Caspase-9; **(D)** P53; **(E)** Bak; **(F)** Bax.

To study the mechanisms of AFB1-induced apoptosis in the liver of broilers, the mRNA expression of BCL-2, Caspase-3, Caspase-9, p53, Bak, and Bax were analyzed in the liver using qRT-PCR on day 14 and 28. The mRNA expression level of BCL2 was down-regulated and the levels of CASP3, CASP9, p53, BAK1, and BAX were up-regulated in the AFB1 contaminated feed group as compared to those in the control group (*P* < 0.05). However, mRNA expression level of Caspase-3 and p53 had no significant difference on day 14 as compared to the control. In contrast, when AFB1 affected birds were treated with different doses of PCPE and positive drug (YCHT), the mRNA expression level of BCL2 was significantly up-regulated and the levels of CASP3, CASP9, p53, BAK1, and BAX were down-regulated on day 28 (Figure 4).

### Serum Immunoglobulins

**Figure 5:**
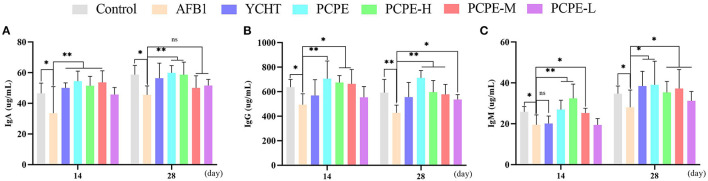
Analysis of serum immunoglobulins on 14^th^ and 28^th^ day of experiment. Asterisk means significant difference (*p* < 0.05) compared to the control and AFB1 group (*P < 0.05; **P < 0.01, ns, non-significant). **(A)** IgA **(B)** IgG **(C)** IgM.

The feeding diet of broilers containing AFB1 (3 mg/kg feed) significantly altered the immune response of birds on day 28 by decreasing serum IgA, IgG and IgM levels significantly as compared with that of the control group (*p* < 0.05). In contrary, the addition of PCPE and positive drug YCHT to AFB1 contaminated diet group significantly alleviated (*p* < 0.05) the toxic effect of AFB1 on serum immunoglobulin levels by increasing the IgA, IgG, and IgM content especially in PCPE alone and high dose groups (Figure 5).

### Effect of PCPE on Growth Performance of Broilers

**Figure 6:**
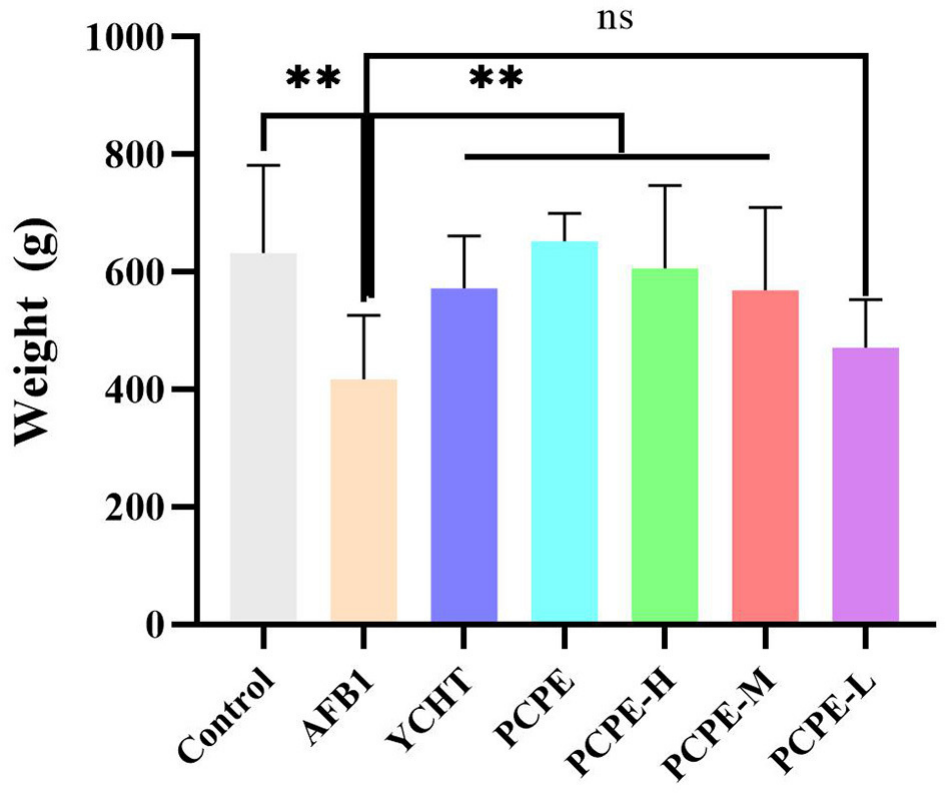
Effect of PCPE on average final weight gain, growth performance of broilers whose feed diet contained AFB1 and PCPE low, medium, and high doses. The average weekly body weight of each group. **P < 0.01.

The effects of AFB1 and PCPE treatments on weekly growth performance are summarized in Figure 6. During the experiment, the AFB1-feed contaminated (3 mg/kg feed) group showed a significantly lower final weight gain and performance with 95% morbidity and 4% mortality as compared with other groups (*p* < 0.05). There was a notable difference in all groups as compared with the AFB1 group. The average weight gain and performance were improved by the addition of PCPE into feed contaminated with AFB1, with a significant increase (*p* < 0.05) in weight gain and performance on day 28 as compared with the AFB1 group (Figure 6).

## Discussion

Poultry industry involves a variety of domestic birds which are susceptible to various toxicants, likewise aflatoxins, and the presence of AFB1 in the poultry environment is a substantial risk to the poultry and its connection to the safety of food products (1). AFB1 has an impact on various biological and physiological functions of poultry birds, including oxidative stress, decreased immunity, inflammation, cell apoptosis, and liver damage (20). The liver is a vital organ responsible for the elimination and detoxification of xenobiotics and toxicants, including AFB1 (21). Earlier reported data speculated that AFB1 involved in damaging and disturbing effects on the structure and function of liver resulted in the disturbance of functional liver enzymes, oxidative stress, tissue damage, apoptosis, and finally organ failure (22, 23). Recently, Penthorum chinense Pursh, a novel folk medicine, is reported for its hepato-protective effects in a variety of pathological problems of the liver, including detoxification, anti-oxidation, promotion of xenobiotics into the blood (24–26), and can also be applied for liver intoxication due to AFB1.

The current research trial demonstrated the positive effects of PCPE in AFB1-challenged liver toxicity in broilers and investigated the mitochondrial apoptosis-concerned pathways linked with PCPE and AFB1 administration. During the current research trial, consequences of AFB1 were disturbances in the functional liver indicators, periportal fibrosis, fatty degeneration, bile duct hyperplasia, and oxidative stress enzymes, which were significantly reversed by the addition of PCPE.

The presence of functional liver indicators in the blood is connected with pathological and physiological changes in the liver (27). Hepatocytes and liver parenchyma are the important cellular components of the liver. Both structures are necessary for a variety of functions, such as metabolic, vascular, immunological, secretory, and eliminatory, along with detoxification of xenobiotics or poisons (28). These structures can be impaired by a continuous exposure of the organ toward toxicants and can result in the liberation of liver enzymes.

A majority of disease pathogenesis occur due to a variety of factors or causes, and oxidative stress is an important one amongst them, that encourages the development of diseases by unbalancing the redox homeostasis of the body. Free radicals and lipid peroxidation mechanisms can be stimulated by the impact of AFB1 and can finally trigger oxidative stress in the liver (29–31). Oxidative stress is regulated by various key components, including antioxidant enzymes (SOD, CAT), MDA, GSH, and GSH-Px. SOD is concerned with the exportation of O2^−^ (32) [30], CAT has a role in cell defense, GSH and GSH-Px play a role in cell resistance, while MDA is an oxidative lipid metabolite that represents cellular injury and lipid peroxidation profile (33). Our outcomes coincide with those of previously reported studies which revealed that AFB1 can cause oxidative stress in the liver of broilers (1, 19). Further underlying mechanisms related with liver damage were validated by the apoptosis signaling pathways.

Apoptosis is important for cellular development as it finally exports the injured cells (34, 35). However, AFB1-related toxicity results in increased apoptosis that leads to liver damage (36–38). Apoptosis is governed by two signaling pathways, extrinsic and intrinsic, which are activated by either mitochondrial signal transduction or receptor-related stimuli (34, 39). It is well established that mitochondria plays an important role in controlling apoptosis as well as repairing eukaryotes for survival (40, 41). The mitochondrial membrane is covered by the BCL2 protein family which interrelates with the tumor suppressor P53. P53 is involved in the catalysis of BAK and encourages transcription-independent stimulation of BAK and BAX. Both the stimulated components are oligomerized on the mitochondrial membrane and result in permeabilization. These pathways are involved in the liberation of pro-apoptotic factors (CYC) from the mitochondria into the cytoplasm, and further stimulation of the caspase cascade family (42, 43). Finally, apoptosis is encouraged by the activation of caspase-9 and then caspase-3, which is due to the signal generated from the inside of mitochondria. In contrary, BCL2 involved in the inhibition of the stimulated BAX and BAK, results in apoptosis reversion (44). In this study, the transcription of *BCL2* was down-regulated and up-regulations were observed in *CASP3, CASP9, p53, BAK1, and BAX* by the addition of AFB1, while PCPE addition in the feed produced opposite effects. These results highlight that AFB1 is involved in the mitochondrial-related apoptosis (42). Thus, PCPE application prevented the liver cells from further activation of apoptosis, by reversing the mitochondrial apoptosis pathway which was triggered by AFB1 toxicant.

During the last five decades it is revealed that aflatoxins have negative effects on chicken performance development of bursa and immunity and research concluded that addition of each mg of AFB1/kg in poultry diet significantly decreased the weight gain and performance of broilers by 5% (45). It is well established that AFB1 toxicants have a bad influence on immunity and immune system is very sensitive to this toxin (46) and growth performance, along with morbidity and mortality in broilers (47, 48). Accordingly, AFB1 followed the same pattern during the current study. However, the addition of PCPE in the diet of broilers resulted in improved immunity as well as growth performance.

## Conclusion

PCPE administration in AFB1-challenged broilers diet had a therapeutic impact on growth and performance and reversed the pathological changes associated with AFB1 in the liver of broilers. Furthermore, PCPE prevented oxidative stress and apoptosis and delimited liver dysfunction and the imbalance in mitochondrial dynamics. Hence, PCPE produced a therapeutic impact on oxidative stress and apoptosis in AFB1-toxoid broilers. PCPE application can be a traditional medicine approach in veterinary clinics while handling mycotoxin-related toxicity in poultry. However, further studies are suggested to understanding of the link between AFB1 and intestinal mucus and permeability in poultry.

## Data Availability Statement

The original contributions presented in the study are included in the article/supplementary material, further inquiries can be directed to the corresponding author.

## Ethics Statement

The animal study was reviewed and approved by all experiment procedures agreed with animal ethics regulations and accepted by the Institutional Animal Care and Use Committee (IACUC) of Southwest University (IACUC-20201203).

## Author Contributions

WT, FN, RY, and JL conceptualized and designed the study. WT and JL analyzed and interpreted the data. WT, ZL, YS, JF, and QL performed the experiments. WT, ZB, and FN wrote the first draft of the manuscript. All authors have contributed in the revision of the manuscript, and have read and approved the submitted version.

## Funding

This research was funded by the Special Project for Fundamental Work of Science and Technology, Grant Number 2013FY110600-03; and special funding for Chongqing Post-Doctoral Research project 2020, number 7820100603.

## Conflict of Interest

The authors declare that the research was conducted in the absence of any commercial or financial relationships that could be construed as a potential conflict of interest.

## Publisher's Note

All claims expressed in this article are solely those of the authors and do not necessarily represent those of their affiliated organizations, or those of the publisher, the editors and the reviewers. Any product that may be evaluated in this article, or claim that may be made by its manufacturer, is not guaranteed or endorsed by the publisher.
